# Extended adjuvant hormonal therapy with exemestane has no detrimental effect on the lipid profile of postmenopausal breast cancer patients: final results of the ATENA lipid substudy

**DOI:** 10.1186/bcr2320

**Published:** 2009-06-16

**Authors:** Christos Markopoulos, Urania Dafni, John Misitzis, Vasilios Zobolas, Evagelos Tzoracoleftherakis, Dimitrios Koukouras, Grigorios Xepapadakis, John Papadiamantis, Basileios Venizelos, Zoh Antonopoulou, Helen Gogas

**Affiliations:** 1 Hellenic Society of Breast Surgeons, 11523 Athens, Greece; 2 Laboratory of Biostatistics, Department of Nursing, University of Athens, Athens, Greece

## Abstract

**Introduction:**

Extended adjuvant endocrine therapy for breast cancer with aromatase inhibitors may potentially alter the lipid profile of postmenopausal patients and thus increase the risk of developing cardiovascular disease. In this study, a subprotocol of the ATENA (Adjuvant post-Tamoxifen Exemestane versus Nothing Applied) trial, we compared the effect of the steroidal aromatase inactivator exemestane on the lipid profile of postmenopausal patients with operable breast cancer, in the adjuvant setting, with that of observation alone after completion of 5 to 7 years of primary treatment with tamoxifen.

**Methods:**

In this open-label, randomized, parallel-group study, 411 postmenopausal patients with operable breast cancer, who had been treated with tamoxifen for 5 to 7 years, were randomized to either 5 additional years of exemestane (25 mg/day; n = 211) or observation only (n = 200). Assessments of total cholesterol (TC), high-density lipoprotein (HDL), low-density lipoprotein (LDL), and total serum triglycerides (TRG) were performed at baseline and then during each follow-up visit, performed at either 6 or 12 months, according to the center's clinical practice, until completing 24 months in the study.

**Results:**

TC and LDL levels increased significantly across time for both arms; TC increase was more pronounced for the observation arm, and that was sustained up to 24 months. HDL levels decreased significantly across time for the exemestane arm, whereas no significant change was detected across time for the observation arm. Triglyceride levels decreased significantly across time on both arms, with no difference detected in changes from baseline between the exemestane and the observation arms.

**Conclusions:**

Exemestane lacks the beneficial effect of tamoxifen on lipids; however, sequential adjuvant treatment with exemestane in postmenopausal breast cancer patients after cessation of 5 to 7 years of tamoxifen does not appear to alter the lipid profile significantly compared with that of an observational arm.

**Trial Registration:**

ClinicalTrials.gov ID: NCT00810706.

## Introduction

The primary objective of the adjuvant hormonal treatment is to reduce risk of recurrence and therefore increase overall survival. In postmenopausal women, the two most commonly used strategies of endocrine treatment are either the interference with estrogen signaling by binding to the estrogen-receptor protein with a selective estrogen-receptor modulator (SERM), such as tamoxifen, or the inhibition of endogenous estrogen production by using an aromatase inhibitor (AI).

Tamoxifen has been the standard adjuvant endocrine therapy for postmenopausal women with breast cancer for more than 30 years; however, its use in recent years has been questioned after indication of an increased risk of endometrial cancer, thromboembolic events, and tolerability concerns [1]. These risks are considered to be a consequence of tamoxifen partial estrogen-agonistic effect. These limitations, along with, most important, the development of resistance, urged the expansion of different approaches in the systemic adjuvant treatment of breast cancer. Notably, fulvestrant, a novel estrogen-receptor antagonist that produces complete receptor blockade and has no estrogen-agonistic activity, is currently licensed only for the treatment of advanced breast cancer after recurrence or progression with prior endocrine therapy [2,3].

More recently, inhibition of aromatase, the enzyme that converts androgens to estrogens, with agents including anastrozole, letrozole, and exemestane, has been shown to be an effective alternative to tamoxifen for postmenopausal women with hormone-dependent breast cancer. Treatment with AIs produces frequent and durable responses in postmenopausal women previously treated with tamoxifen or endocrine ablative surgery, and AIs are more effective than tamoxifen in producing responses and delaying progression in first-line treatment of metastatic disease [4,5].

Clinical trials established the role of AIs in the adjuvant therapy for postmenopausal women with hormone-responsive breast cancer in upfront, switch, and sequential treatment settings [6-9], and this is reflected by international guidelines such as the American Society of Clinical Oncology [10], St. Gallen [11], National Comprehensive Cancer Network [12], and others.

The EBCTCG group was recently shown, through an overview of the randomized trials, that more than 50% of disease recurrences occur after the end of 5 years of tamoxifen treatment, possibly because of micrometastatic disease that may still be present [13]. Extending tamoxifen use further than the standard 5-year duration of treatment has been proven to be ineffective [14]. Postmenopausal women with hormone receptor-positive tumors who have completed about 5 years of adjuvant tamoxifen therapy should be considered for treatment with an AI. This strategy has been widely studied by the National Cancer Institute of Canada MA.17 [9], the Austrian Breast and Colorectal Cancer Study Group (ABCSG) 6a [15], and the NSABP B-33 [16] trials. The MA.17 trial was the first to demonstrate that an AI in the extended adjuvant-treatment setting was effective in reducing the risk for recurrence, and the first results from ABCSG-6a and NSABP-33 further supported the effectiveness of this treatment strategy.

However, a question exists on the long-term safety of these agents, in particular with respect to the effects on the lipid profiles of postmenopausal women. Lipid-metabolism disorders can be the cause of a wide range of conditions, with cardiovascular disease being the most significant [17,18]. Cardiovascular disease is the leading cause of death in the developed world for women [19]. The role of low-density lipoprotein cholesterol (LDL) in the pathogenesis of atherosclerosis and subsequently in coronary heart disease is well known. Evidence suggests that increased levels of LDL are highly correlated with increased risk of heart disease, even while total cholesterol remains within normal range [20]. At the same time, high-density lipoprotein (HDL) cholesterol is known for its protective effect against coronary heart disease [21]. The role of triglycerides is less clear, but increased levels have been associated with risk of cardiovascular diseases in both women and men [22].

The risk of cardiovascular mortality increases dramatically in women after menopause [22] because of lipid-metabolism alterations that are attributed to estrogen deprivation. Levi *et al*. [23] suggested that the greatest cause of death in women with early-stage breast cancer is heart disease. Because of the high levels of estrogen deprivation caused by aromatase inhibitors, the effect of such inhibition on lipid profiles and thus cardiovascular disease [24-26] has been a concern, especially considering the protective effects that tamoxifen exerts on lipid profiles [27,28]. It is, therefore, necessary to study these effects to assess efficiently the cost-to-benefit relation. However, we note that, in the extended adjuvant treatment setting, the effect of aromatase inhibition on lipids should be compared with the post-tamoxifen-deprivation lipid profile. The tamoxifen beneficial effect on the lipid profile of postmenopausal breast cancer patients seems to be lost in less than 12 months after cessation of tamoxifen treatment, and patients are assumed to return within the ranges of the average postmenopausal female population [29].

Exemestane is an irreversible steroidal inhibitor of aromatase [30,31] that was recently shown to confer both an overall and a disease-free survival advantage when given after 2 to 3 years of tamoxifen compared with the standard 5 years of tamoxifen in the adjuvant treatment of postmenopausal breast cancer patients [8,32]. The Adjuvant post-Tamoxifen Exemestane versus Nothing Applied (ATENA) trial was an open-label randomized parallel-group study of postmenopausal women with operable breast cancer who had been treated with tamoxifen for 5 to 7 years and then switched to exemestane or observation alone for 5 additional years. The ATENA lipid substudy compared alterations in the lipid profile after initiation of exemestane treatment with those of women who cease tamoxifen treatment and thus lose the beneficial effect of tamoxifen on lipids. Preliminary results have already been published [33]. Here we report the analysis of the final results at 24 months of treatment.

## Materials and methods

The ATENA phase III randomized parallel-group multicenter trial was designed to compare 5 years of adjuvant exemestane with 5 years of observation in postmenopausal women with operable breast cancer who had received 5 to 7 years of adjuvant tamoxifen. Recruitment of 1,803 core patients was planned in the ATENA trial from study sites of the Hellenic Breast Surgeons Society. The primary end point for the core protocol was disease-free survival (DFS). The lipid substudy was designed to evaluate changes in the patients' serum lipid profile during study treatment. The ATENA trial was prematurely discontinued because of the publication of the MA.17 trial results [9]. A total of 411 of 448 patients randomized in the ATENA trial until closure were eligible for the lipid substudy. Exemestane treatment (25 mg/day) was planned for 5 years, unless disease relapse or excessive toxicity was documented, the patient refused further treatment, or any new anticancer therapy was initiated.

All patients entering the substudy had no history of any concomitant disease that could affect the lipid profile, including familial dyslipidemia. No reports of cholesterol-lowering agent consumption existed in our concomitant-medication database.

Patients were not instructed to follow a specific diet during the study; however, before offering a blood sample, all patients were required to fast for 12 hours. Blood samples for lipid-profile analysis (cholesterol, HDL, LDL, and total triglycerides) were measured at baseline and then during each follow-up visit, performed at either 6 or 12 months, according to the standard clinical practice at each participating centre.

The study was approved by the relevant local institutional ethics committees and was conducted according to the Declaration of Helsinki. Informed-consent forms were accordingly signed by all patients before entering randomization.

### Statistical analysis

Descriptive statistics (mean, standard deviation) by treatment group of all lipid variables and of corresponding differences from baseline are presented for each visit. Mixed-effects models were used for estimating the time and treatment effects on each of the four lipid parameters for the study duration. Compound symmetry variance-covariance matrix and a random intercept effect were used in these models. These polynomial growth-curve models reliably explore trends across time and between treatment groups, by taking into account the within-subject variability and the common problem, in repeated-measures data, of missing values [34]. The mixed-effects models were run both on the actual lipid parameter values and on their logarithms. Estimations of absolute value means and mean changes from baseline for each treatment arm at each time point obtained from the appropriate mixed-effects model are presented in tables and figures. Analysis was performed by using the SAS statistical package (SAS Institute Inc., Cary, NC, USA). All reported *P *values are two-sided, and results were considered significant at α = 0.05.

## Results

Four hundred forty-eight patients were randomized in the ATENA trial from January 2001 until premature closure of the trial on November 2005 due to poor recruitment, as a result of the publication of the MA.17 trial results. Two recruiting centers did not participate in the lipid substudy, and 14 patients were not eligible because of consumption of cholesterol-lowering agents. In total, 411 of 448 patients randomized in the ATENA trial were eligible for the lipid substudy, with 211 patients randomized to the exemestane arm (E), and 200 patients, to the observation arm (O). This is the final report on the analysis of data during the 2-year study period.

Patient characteristics, including age, weight at randomization, prior adjuvant chemotherapy or radiotherapy, and ECOG performance status are presented in Table 1. No significant differences were found between the two arms for any baseline parameter. At 12 months, 162 patients had available measurement for cholesterol level (90 in E and 72 in O), 107 for HDL (59 in E, 48 in O), 97 for LDL (53 in E, 44 in O), and 144 for triglyceride levels (84 in E and 60 in O). The corresponding sample sizes at 24 months were 94 for the cholesterol level (45 in E, 49 in O), 64 for HDL (27 in E, 37 in O), 64 for LDL (26 in E, 38 in O) and 86 for Triglyceride levels (43 in E, 43 in O).

**Table 1:** Patient characteristics

		Exemestane	Observation	Total
Patients	Number	211	200	411

Age	Mean	62.6	61.8	62.2
	(range)	(40–81)	(39–81)	(39–81)
Weight	Mean	71.8	69.6	70.6
	(range)	(48–100)	(52–100)	(48–100)
Adjuvant chemotherapy	*n *(%)			
No		131 (62.09)	121 (60.5)	252 (61.31)
Yes		80 (37.91)	79 (39.5)	159 (38.69)
Local radiation therapy	*n *(%)			
No		77 (36.49)	82 (41.0)	159 (38.69)
Yes		117 (55.45)	99 (49.5)	216 (52.55)
Missing data		17 (8.06)	19 (9.5)	36 (8.76)
ECOG performance status	*n *(%)			
0		164 (77.73)	148 (74.0)	312 (75.91)
1		33 (15.64)	41 (20.5)	74 (18.0)
2		1 (0.47)	0 (0.0)	1 (0.24)
Not reported		13 (6.16)	11 (5.5)	24 (5.84)
ER and PgR status	*n *(%)			
ER^+^/PgR^+^		110 (52.13)	107 (53.5)	217 (52.8)
ER^+^/PgR^-^		23 (10.9)	19 (9.5)	42 (10.22)
ER^-^/PgR^+^		13 (6.16)	10 (5.0)	23 (5.6)
ER^+^/PgR unknown		15 (7.11)	11 (5.5)	26 (6.33)
ER and PgR unknown		50 (23.7)	53 (26.5)	103 (25.06)

Mean observed absolute values and corresponding standard deviations (SDs) for each lipid parameter at baseline, at 1 year, at 1.5 years, and at 2 years after baseline are presented in Table 2, whereas mean observed changes from baseline values over the study period are presented in Table 3. The corresponding differences between treatments of mean absolute values over the study period, as estimated from the mixed-effects models, are presented in Table 4.

**Table 2:** Observed absolute values for lipid parameters [mg/dl] across the study period (mean ± standard error)

	Cholesterol value		HDL value		LDL value		Total triglycerides value	
	E	O	E	O	E	O	E	O
Baseline	215 ± 3.4	217 ± 3.3	57 ± 1.3	57 ± 1.4	136 ± 4.5	133 ± 4.2	136 ± 6.1	135 ± 5.8
6 mo	227 ± 4.2	226 ± 5.8	56 ± 1.6	60 ± 1.5	149 ± 4.5	143 ± 7.3	116 ± 7	127 ± 7.8
12 mo	228 ± 4.2	231 ± 4.5	53 ± 1.4	60 ± 1.7	152 ± 4.9	145 ± 4.3	111 ± 5.5	117 ± 6
18 mo	234 ± 6	228 ± 6	51 ± 1.8	60 ± 2.1	161 ± 7.7	145 ± 6.8	121 ± 6.5	111 ± 9
24 mo	228 ± 5.6	230 ± 4.8	52 ± 1.9	62 ± 1.9	154 ± 6.3	136 ± 6.5	112 ± 6.5	118 ± 7.8

E = Exemestane; O = Observation.

**Table 3:** Observed changes from baseline values over the study period (mean ± standard error)

		6 mo		12 mo		18 mo		24 mo	
		Mean ± SEM	*P*	Mean ± SEM	*P*	Mean ± SEM	*P*	Mean ± SEM	*P*

Cholesterol change	E	11.5 ± 5.6	0.046	14.4 ± 5	0.005	15.3 ± 7	0.037	8.9 ± 7.4	NS
	O	16.8 ± 4.7	0.001	21.6 ± 4.1	<0.001	24 ± 5.3	<0.001	17 ± 6	0.009
HDL change	E	-4.3 ± 1.8	0.025	-3 ± 2.3	NS	-6.5 ± 2.6	0.024	-8.3 ± 2.1	0.001
	O	2.2 ± 1.5	NS	1.7 ± 1.4	NS	1.5 ± 1.2	NS	1.1 ± 1.9	NS
LDL change	E	24.3 ± 6.3	0.001	20.1 ± 6.3	0.004	22.7 ± 9.5	0.030	32.1 ± 8.1	0.001
	O	19.8 ± 5.7	0.002	22.3 ± 4.9	<0.001	30.4 ± 6.2	<0.001	23 ± 8.4	0.015
Total TRG change	E	-24.1 ± 7.8	0.004	-32.2 ± 7.6	<0.001	-26.8 ± 9.3	0.008	-20.7 ± 9.6	0.040
	O	-13.7 ± 6.1	0.031	-17.6 ± 7.6	0.026	-9.4 ± 7.2	NS	-19.8 ± 9	0.038

E = exemestane; O = observation.

Note: *t*-test *P *values, NS = nonsignificant when *P *> 0.05.

**Table 4:** Estimated differences between treatments of mean absolute values for lipid parameters across the study period according to the mixed-effects model (mean ± standard error).

		Cholesterol value	HDL value	LDL value	Total TRG value
		E-O^a^	E-O	E-O^a^	E-O^a^
Baseline	Mean ± SEM		-0.25 ± 1.36		
	*P*		0.85		
6 months	Mean ± SEM		-2.96 ± 1.07		
	*P*		0.01		
12 months	Mean ± SEM	-1.23 ± 2.93	-5.68 ± 1.35	7.77 ± 4.42	-0.02 ± 0.03
	*P*	0.68	<0.0001	0.08	0.50
18 months	Mean ± SEM		-8.39 ± 1.97		
	*P*		<0.0001		
24 months	Mean ± SEM		-11.1 ± 2.7		
	*P*		<0.0001		

E = exemestane; O = observation.

^a^Note: Constant difference between E and O across time.

**Figure 1:**
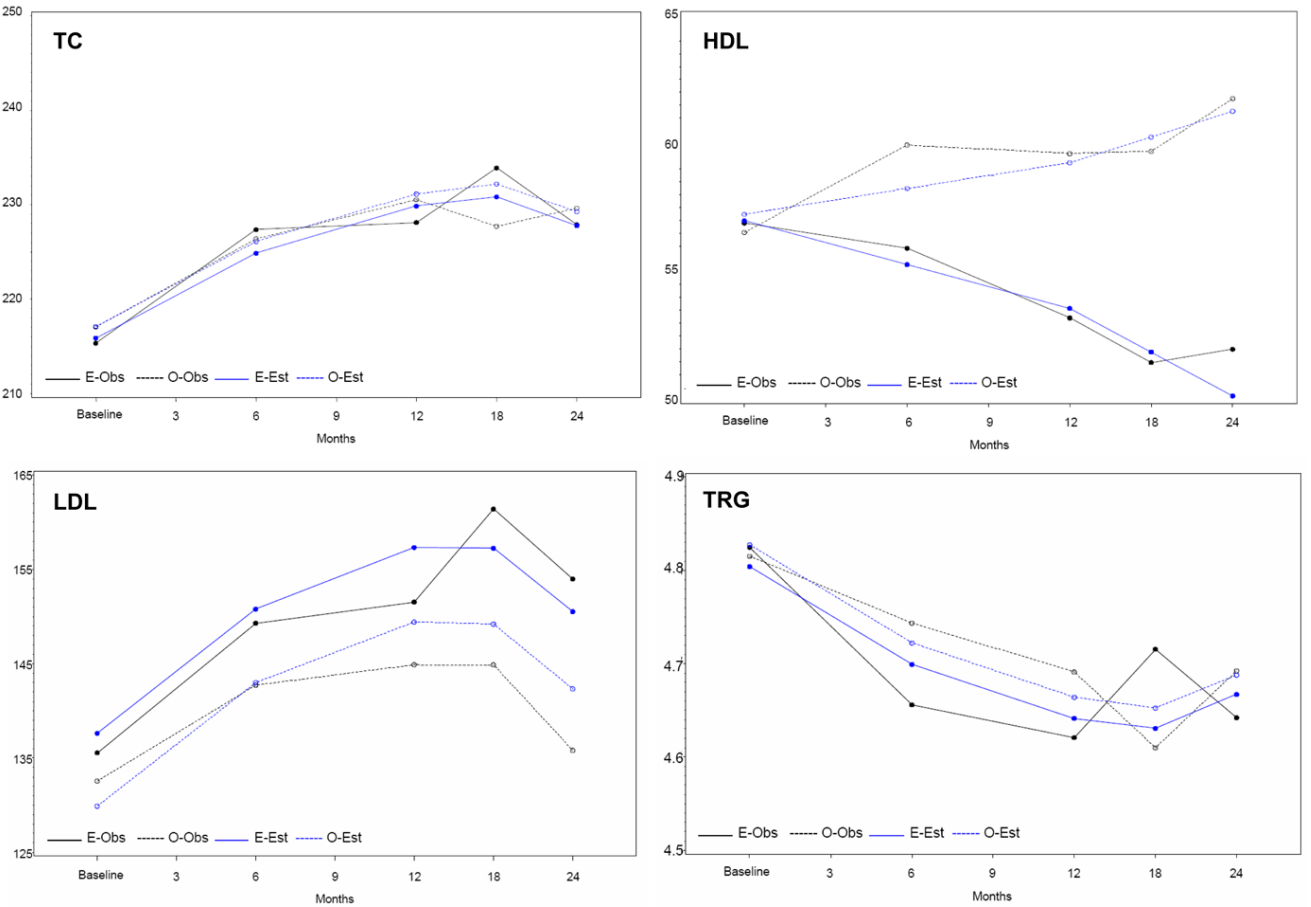
Estimated mean values versus observed for total cholesterol (TC), high-density lipoprotein (HDL), low-density lipoprotein (LDL), and triglycerides (total log TRG).

Figure 1 shows, by treatment arm, the observed and the estimated mean values for each lipid parameter across time.

Cholesterol for the first 18 months increased significantly across time for both arms, an effect that is more pronounced for the observation arm and is sustained up to 24 months (*P *= 0.0005, *P *= 0.019 for time and time-squared effect, respectively; Tables 2 through 4). The corresponding estimated differences between the E and O arms in absolute changes from baseline were not significant across time (Table 4 and Figure 1).

The HDL levels decreased significantly across time for the exemestane arm, whereas an increase was detected across time for the observation arm, without reaching statistical significance (*P *= 0.08; Tables 2 through 4, Figure 1). The effect of time was significantly different between treatment arms (treatment group × time interaction, *P *value = 0.0012), with the mean absolute HDL levels for the observation arm being consistently higher across time than for the exemestane arm, and the distance between them increasing with time (Table 4 and Figure 1).

The LDL levels increased significantly across time (time *P *value < 0.0001) similarly for both arms (treatment *P *value = 0.08), with an initial sharp increase diminishing up to the 18-month time point, at which a trend reversal leading to smaller mean values at 24 months is apparent (time-squared *P *value < 0.0001; Tables 2 through 4). The corresponding estimated mean differences between the E and O arms in absolute changes from baseline were constant across time, equal to 7.77 ± 4.42 mg/dl (Table 4 and Figure 1).

Triglyceride levels decreased significantly across time on both arms, with no difference detected in changes from baseline between E and O (Tables 2 through 4). Observed values show a variable behavior, increasing or decreasing in adjacent time points. The appropriate polynomial mixed-effect model was fit on the logarithm of triglyceride levels (*P *= 0.0005, *P *= 0.019 for time and time squared, respectively). In Figure 1, the observed and the model-based estimated absolute values of triglyceride levels are presented in the logarithmic scale.

## Discussion

It is generally accepted that adjuvant hormonal treatment with tamoxifen has a beneficial effect on serum lipids of postmenopausal breast cancer patients, which is possibly due to its agonistic estrogenic activity. Conversely, the use of AIs has raised some concerns with respect to lipid profiles because of estrogen deprivation caused by aromatase inhibition. However, considering that exemestane is an irreversible steroidal AI requiring *de novo *enzyme synthesis for estrogen synthesis, exemestane and its metabolites have been suggested to have a protective effect on lipid metabolism compared with the nonsteroidal aromatase inhibitors [35-37]. The effect of extending treatment with exemestane after several years of adjuvant tamoxifen does not appear to differ significantly from the effect of tamoxifen deprivation itself, in terms of lipid profile, indicating that exemestane is not adversely detrimental to lipid parameters when compared with observation alone.

Our final findings from the ATENA lipid substudy confirm the protective effect of tamoxifen on the lipid profile and indicate an overall trend for increasing cholesterol levels after cessation of tamoxifen in the whole population. Total cholesterol levels increased significantly in both arms, with a more-pronounced increase in the observation arm, whereas overall, no difference was found between the two groups. HDL levels in the exemestane arm seemed to decrease significantly after the first year of treatment, an effect that was different from that observed in our preliminary analysis at the end of the 1-year study period. The initial sharp increase in LDL cholesterol, as it was reported during our initial report, seems to smooth out to a smaller increase at 24 months for both groups, whereas consistency was noted in the finding of lack of significance between the two treatment groups. Our final results also confirm the significant decrease across time in both arms for triglyceride levels. Evidence was noted that lower TRG levels below the 'threshold' value of about 1.5 mmol/l (133 mg/dl) will cause a change in the LDL-size profile to larger, less dense and, therefore, less atherogenic species [38]. In this context, the statistically significant increase in the LDL levels observed at 12 months in the group of patients treated with exemestane should be considered of lesser clinical importance because it is accompanied by a significant decrease in the TRG levels.

It is rather difficult to assess the impact of AIs on the lipid profile of postmenopausal patients by comparing results of published studies because of the different designs of trials. Tamoxifen was the comparator in most major studies, with the exception of MA17, in which letrozole was compared with placebo [9]. Additionally, the results depend on the duration of therapy and on how the lipid data were collected. However, our final findings are overall in accordance with results from other previously published trials regarding the effect of AIs on lipid profiles.

In a study by Engan *et al*. (39), which examined the lipid profile in patients receiving exemestane or tamoxifen, 12 postmenopausal metastatic breast cancer (MBC) patients received escalating doses of exemestane (5 to 200 mg/day), and 6 patients received 20 to 30 mg/day tamoxifen; significant reductions in TC, TRG, HDL, and Apo A1 levels were observed after 12 weeks of treatment with exemestane. TC was significantly decreased after 9 weeks of tamoxifen treatment [39]. In another prospective, randomized phase II study, the EORTC trial 10951, examining serum lipid profiles in postmenopausal MBC patients, Atalay *et al*. (40), reported that exemestane and tamoxifen had opposite effects on TRG levels: exemestane reduced whereas tamoxifen increased TRG levels over time. In their study of 122 women, lipid parameters (TC, TRG, HDL, Apo 1, Apo B, and Lip a) were monitored at 8, 24, and 48 weeks of treatment. Apart from TRG and Apo A1, no other lipid parameters were altered during the study for either treatment arm. The authors concluded that because their population was relatively small and many patients did not have data at the later time points, a larger population should be studied to confirm the results. Hypercholesterolemia was not reported in the IES trial of sequential exemestane after tamoxifen [8,32].

In another study examining the lipid profiles in 55 postmenopausal women with early breast cancer who switched to exemestane after at least 2 years of tamoxifen treatment, triglycerides and HDL cholesterol significantly decreased in the exemestane group, whereas LDL cholesterol significantly increased at the end of the 1-year study period [41]. Those results are consistent with ours observed in the 12-month analysis of our data; however, the different duration of our study gives the opportunity for observations for a longer period when the effect of the foregone tamoxifen treatment is lost (29). The sharp increase in LDL levels observed during the first year of the study, similar for both arms, seems to smooth out to smaller mean values during the second year (Table 4 and Figure 1), and it is most likely to represent LDL changes in the lipid profiles of healthy postmenopausal women, whereas the diverse change of HDL levels across time in the two arms of the study (Figure 1) is indicative of a trend for decreased HDL levels in patients with long-term exemestane treatment.

In a placebo-controlled study involving 147 postmenopausal women with early breast cancer, exemestane had no major effect on the lipid profile except for a modest but significant decrease from baseline in HDL cholesterol (*P *< 0.001) and apolipoprotein A1 [42]. Results from the TEAM (Tamoxifen and Exemestane Adjuvant Multicenter) trial lipid substudy conducted by our group suggested a trend for a reduction in total cholesterol in both treatment arms, whereas TC and LDL consistently and significantly decreased in the tamoxifen arm only. The mean HDL level was higher for the tamoxifen arm compared with the exemestane arm across time. No significant trend was detected throughout the study period on triglyceride levels in either arm [43,44].

In terms of the other two extended adjuvant AI trials, our findings are consistent with those of the letrozole MA.17 lipid substudy results. According to this, letrozole had no significant effect on the lipid profiles of 347 postmenopausal women after 36 months of treatment. Both treatment arms experienced increases in total cholesterol, LDL, and triglycerides at 6 months, but this could be perceived as a consequence of the discontinuation of tamoxifen. Levels later leveled off and remained fairly stable [45]. In an extension of the MA.17 study currently under way, patients are being rerandomized to an additional 5 years of letrozole or placebo. Data from this group could allow further investigation of the lipid and cardiovascular effects that may occur with long-term estrogen deprivation.

Adjuvant trials comparing aromatase inhibitors with tamoxifen have also provided evidence of the effect of AIs on lipid metabolism. When comparing tamoxifen therapy with each AI results in some changes in lipid parameter levels, but again, no detrimental effects have been observed. These effects could be due to the beneficial effect of tamoxifen mainly and, to a lesser extent, to the adverse effect of AI therapy.

Initial results of the LEAP trial directly compare safety parameters between the steroidal AI exemestane and the nonsteroidal AIs anastrozole and letrozole in 90 healthy postmenopausal women [46]. Initial results show that no significant differences exist between anastrozole and letrozole in effects on LDL/HDL ratios, triglyceride concentrations, and non-HDL concentrations. Exemestane was associated with an increase in the LDL/HDL ratio (+17) (*P *= 0.047) compared with anastrozole. No median change from baseline is seen in total serum cholesterol for letrozole, a slight increase for anastrozole (+0.4), and a nonsignificant decrease for exemestane (-3.9) (*P *= 0.164 vs. anastrozole).

Current data do not allow drawing any clear conclusions about the effects of AIs on lipid metabolism. However, one must note that AIs lack the cardioprotective effect of tamoxifen. Moreover, most of the trials published today provide data for a follow-up to a maximum of 3 years of treatment. No clear evidence is found of the long-term effect of AIs on lipid parameters and subsequently on cardiovascular health as a whole. However, the benefits of receiving an AI are likely to outweigh the risk of the effect on lipid profiles.

## Conclusions

Our results are in agreement with those of previously published AI lipid trials. Exemestane does not have the protective effect of tamoxifen on lipids. A trend for decreased HDL levels was noticed across time in patients with long-term exemestane treatment. However, extended adjuvant treatment with exemestane in postmenopausal breast cancer patients after cessation of 5 to 7 years of tamoxifen does not appear overall to alter significantly the lipid profile compared with that of an observational arm.

## Abbreviations

ABCSG: Austrian Breast and Colorectal Cancer Study Group; AI: aromatase inhibitor; ATENA: Adjuvant post-Tamoxifen Exemestane versus Nothing Applied trial; E: exemestane; EBCTCG: Early Breast Cancer Trialists' Collaborative Group; ECOG: Eastern Cooperative Oncology Group; HDL: high-density lipoprotein; HRT: hormone replacement therapy; LEAP: The Letrozole, Exemestane and Anastrozole Pharmacodynamics trial; LDL: low-density lipoprotein; MBC: metastatic breast cancer; NSABP: National Surgical Adjuvant Breast and Bowel Project; O: observation; SERM: selective estrogen-receptor modulator; TC: total cholesterol; TEAM: Tamoxifen and Exemestane Adjuvant Multicenter trial; TRG: total serum triglycerides.

## Competing interests

Professor Markopoulos has received educational grants and honoraria for lectures from AstraZeneca, Novartis, and Pfizer. All other authors have received in the past unrestricted educational grants from AstraZeneca, Novartis, and Pfizer, with the exception of Dr. Dafni, who declares no conflict of interest.

## Authors' contributions

CM conceived, designed, and coordinated the study, provided study material, and drafted the manuscript. UD coordinated the study, performed the statistical analysis, and helped to draft the manuscript. JM, VZ, ET, DK, GX, JP, BV, and ZA participated in the design of the study and provided study material. HG participated in the design of the study, provided study material, and helped to draft the manuscript. All authors read and approved the final manuscript.
